# Left ventricle inflow and outflow tract angle in normal fetuses

**DOI:** 10.3389/fcvm.2023.1257475

**Published:** 2023-11-24

**Authors:** Yang Yang, Ran Xu, Heyi Tan, Dan Zhou, Jiawei Zhou, Shi Zeng

**Affiliations:** ^1^ Department of Ultrasound, Second Xiangya Hospital, Central South University, Changsha, China; ^2^ Department of Urology, Second Xiangya Hospital, Central South University, Changsha, China

**Keywords:** fetus, fetal cardiovascular, fetal echocardiography, mid-third trimester, left ventricle inflow and outflow angle

## Abstract

**Objective:**

Impaired elasticity of aorta has been observed in fetuses with congenital cardiac disease, while the orientation of left ventricle outflow tract has been found to influence the blood flow in the ascending aorta. Therefore, the objective of this study is to examine the left ventricle inflow and outflow tract angle (LIOA) in healthy fetuses.

**Method:**

A total of 668 fetuses were enrolled in this prospective study. The LIOA were measured with two-line method at left ventricle inflow and outflow tract view. Pearson's correlation coefficient was utilized to assess the associations between LIOA and estimated fetal weight (EFW) and cardiac dimensions, including cardiac axis and diameters of aortic valve (AV), pulmonary artery valve (PAV), mitral valve (MV) and tricuspid valve (TV).

**Results:**

The LIOA was determined to be 44 ± 7.5^°^ (mean ± SD). No significant difference was observed in the LIOA across different gestational ages (GAs). A mild positive correlation was observed between LIOA and cardiac axis. However, no significant associations were found between LIOA and parameters such as EFW, as well as diameters of AV, PAV, MV and TV.

**Conclusion:**

The LIOA remained constant during the mid-third trimester and was mildly positively correlated with cardiac axis in normal fetuses.

## 1. Introduction

Impaired elasticity of ascending aorta has been demonstrated in fetuses with congenital heart defect (CHD). Our previous studies have showed decreased aortic strain (1), circumferential strain, fractional area change, and longitudinal strain (2) in the ascending aorta of fetuses with coarctation of aorta (CoA) as well as impaired aortic elastic properties in those with tetralogy of Fallot (TOF) (3). The underlying mechanism responsible for this impaired aortic elasticity in CHD fetuses is not yet fully understood. In addition to the significant histological abnormalities found in the ascending aorta (4, 5), hemodynamic disturbance caused by the inherent ascending geometry in CHD (6) may be an important contributing factor to the development of aorta elasticity.

The angel between LV inflow and outflow tract, known as the left ventricle inflow and outflow tract angle (LIOA), plays a crucial role in maintaining a normal blood flow pattern in the ascending aorta. Kauhanen et al. described this angle as the “heart-aortic angle” in adults with coronary artery disease using computed tomography angiograms. They found a smaller “heart-aortic angle” with increased total wall shear stress in the outer curvature of the proximal ascending aorta, and it was significantly associated with ascending aorta dilation (7). Besides, the curvature of ascending aorta impacted the blood flow patterns as well. Salmasi et al. found patients with left ventricular outflow tract aortic angles greater than 60^o^ had marked asymmetric flow acceleration on the outer curvature in the proximal aorta. And higher angles associated with higher wall shear stress in the outer curve of the aorta (8). We hypothesized that this angle may also influence the elasticity of the aorta in fetal heart, but little is currently known about LIOA in fetuses. Therefore, the aim of this study was to document the normal LIOA in mid and third trimester fetuses and explore its relations with cardiac dimensions and fetal biometry.

## 2. Material and method

This prospective cross-sectional study was conducted in the Second Xiangya Hospital of Central South University in China. The study protocol received approval by the Ethics Committee of the Second Xiangya Hospital, and the written informed consent was obtained from all participating families. Recruitment of pregnant women took place at the Second Xiangya Hospital from January 2021 to January 2023.

The inclusive criteria for this study were as follows: (a) singleton pregnancy; (b) accurate determination of gestational age (GA) based on factors such as past menstrual regularity, precise recording of the last menstrual period, and ultrasound estimation in the first trimester consistent with the actual GA.

The exclusive criteria were as follows: (a) multiple pregnancy; (b) identifiable chromosomal abnormality; (c) cardiac or extracardiac malformations; (d) persistent fetal arrhythmia; (e) maternal complications that might change fetal hemodynamics, such as gestational diabetes, pregnancy-induced hypertension, preeclampsia, anemia, or thyroid disease; (f) fetal size outside the normal range for GA, defined as estimated fetal weight (EFW) below the 10th percentile or above the 90th percentile for fetuses of the same GA.

The obstetrical ultrasound and echocardiography were performed routinely for each fetus by a single operator using Voluson E8 or E10 system (GE Healthcare, Milwaukee, WI, USA) equipped with C2-9-D and C1-5 probes. Fetal biometry, including the biparietal diameter, head circumference, abdominal circumference, and femoral length, was measured and used to calculate the EFW (9). A standard fetal echocardiogram was conducted by an expert, who measure the dimensions of cardiac chambers and valves at their maximal size following the inner-to- inner edge model. These measurements were then automatically converted to *z*-scores based on GA (10). The mitral valve (MV) and tricuspid valve (TV) were obtained in the 4-chamber view (4CV) during diastole, while the aortic valve (AV) and pulmonary artery valve (PAV) were obtained in longitudinal view during systole.

**Figure 1:**
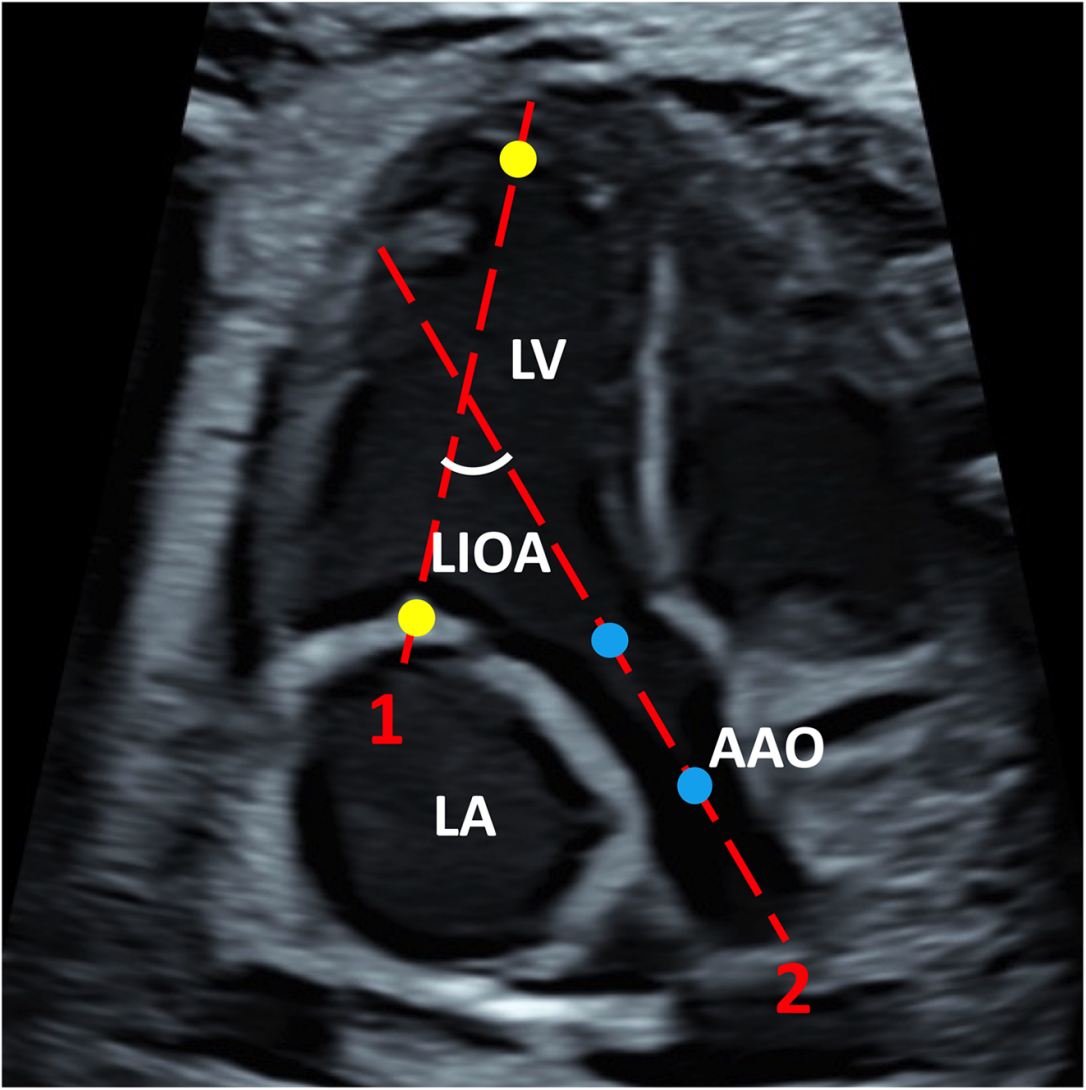
The representative image of LIOA measurement. The LIOA was measured at left ventricular inflow and outflow tract view. Line 1 was positioned from the middle of the mitral valve to the apex which were presented with yellow dots representatively. Line 2 was positioned from the center of aortic valve to the center of the ascending aorta which were presented with blue dots representatively. The acute angle between line 1 and 2 was recorded as LIOA. (LV, left ventricle; LA, left atrium; AAO, ascending aorta; LIOA, left ventricular inflow and outflow angle).

The LIOA was measured at view of left ventricular inflow and outflow tract using the two-line method in systole. Briefly, the image was enlarged until the heart occupied two-thirds of the image with the apex preferably positioned upwards. The first line, indicating LV inflow tract, was positioned from the middle of the mitral valve to the apex. The second line, indicating LV outflow tract, was positioned from the center of aortic valve to the center of the ascending aorta. The acute angle formed between these two lines was recorded as LIOA (Figure 1). Additionally, the angle of fetal cardiac axis was measured using the two-line method at 4CV (11).

Continuous variables were presented as mean ± standard deviation (SD). To compare differences in the fetal LIOA among different GA, a one-way analysis of variance (ANOVA) was conducted. Pearson's correlation coefficient was utilized to examine the correlations between the fetal cardiac axis, EFW, diameters of AV, PAV, MV and TV and LIOA angle. The intra-observer and inter-observer agreement on LIOA was assessed using the intraclass correlation coefficient based on 30 randomly selected observations. A *p*-value of <0.05 was considered statistically significant. All statistical analyses were performed by GraphPad Prism 9 (GraphPad Software, Inc.).

## 3. Result

### 3.1. General condition of fetuses

A total of 691 fetuses, ranging in GA from 16 to 39 weeks, were initially included in this study. Out of these, 14 fetuses were excluded due to poor images quality and nine fetuses were lost to follow up, resulting in a final enrollment of 668 fetuses. The clinical characteristics of this cohort are summarized in Table 1.

**Table 1:** The clinical characteristics of this cohort.

Maternal	
Age, year	32 ± 4
BMI, kg/m^2^	26.2 ± 5.7
Nulliparous	68%
Fetal	
GA at scan, weeks	28 ± 6.1
EFW at scan, g	1,364 ± 931
MV, mm	8.9 ± 2.3
TV, mm	9.2 ± 2.5
AV, mm	4.6 ± 1.1
PAV, mm	5.6 ± 1.3
Cardiac Axis, ^°^	40 ± 7.7
Delivery outcome	
Vaginal delivery, *n*	87%
GA at delivery, weeks	39.3 ± 3.5
<37 weeks, n	3.1%
Birth weight, g	2,870 ± 378
<10th centile, *n*	0
5-min Apgar score < 7, %	0
NICU, %	0

Data are presented as the mean ± SD or frequency (percentage).

BMI, body mass index; EFW, estimated fetal weight; GA, gestational age; MV, mitral valve; TV, tricuspid valve; AV, aortic valve; PAV, pulmonary artery valve; NICU, neonatal intensive care unit.

### 3.2. The LIOA value of fetuses in different Ga

The LIOA was successfully obtained from all fetuses, with the value of 44 ± 7.5^°^ (mean ± SD). Table 2 presents the LIOA values for each GA from 16 to 39 weeks. By comparing the LIOA among different GAs, we observed no significant difference during the mid and third trimester (*P* > 0.05).

**Table 2:** The LIOA at different GA (mean ± SD).

GA	*n*	mean ± SD	GA	*n*	mean ± SD	GA	*n*	mean ± SD
16 weeks	15	49.2 ± 5.1	25 weeks	11	41.7 ± 5.7	33 weeks	17	41.5 ± 6.7
18 weeks	15	42.3 ± 10.7	26 weeks	16	38.9 ± 3.6	34 weeks	26	45.7 ± 8.2
19 weeks	13	51.1 ± 2.1	27 weeks	11	45.2 ± 5.2	35 weeks	15	47.3 ± 8.4
20 weeks	26	46.9 ± 4.6	28 weeks	11	43.6 ± 5.5	36 weeks	11	43.9 ± 5.5
21 weeks	21	45.5 ± 2.4	29 weeks	25	37.5 ± 3.2	37 weeks	37	43.8 ± 7.2
22 weeks	57	41.8 ± 5.2	30 weeks	44	45.4 ± 9.2	38 weeks	10	48.1 ± 8.7
23 weeks	119	44.7 ± 8.1	31 weeks	98	44.1 ± 9.1	39 weeks	11	41.6 ± 7.2
24 weeks	35	47.5 ± 4.4	32 weeks	24	46.3 ± 6.7			

### 3.3. The correlation between LIOA and cardiac axis

**Figure 2:**
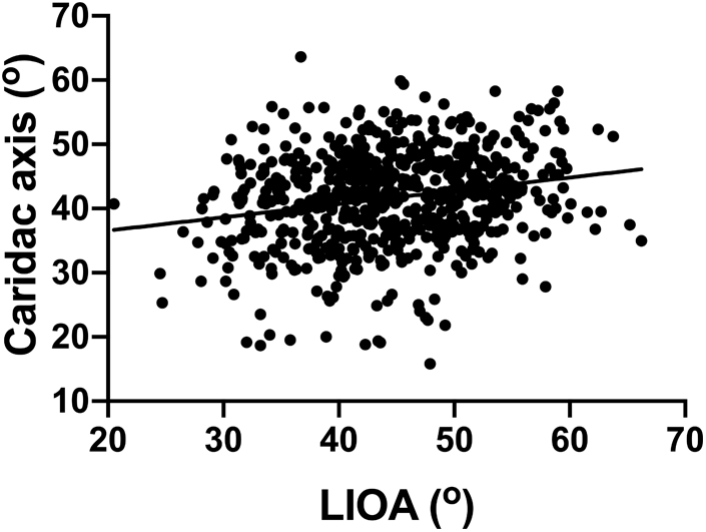
Positive correlation found in the LIOA and cardiac axis. The correlation between the LIOA and cardiac axis was analyzed using Pearson's correlation coefficient with the *r* and *p*-value as 0.22 and <0.0001, respectively.

The cardiac axis from the same group of fetuses was also documented, with value of 40 ± 7.7^°^ (mean ± SD) (Table 1). There was no significant difference observed in the cardiac axis from 16 to 39 weeks which is consistent with previously published data (2, 3). Furthermore, the Pearson analysis suggests a mild positive correlation between cardiac axis and LIOA (*r* = 0.22, *p* < 0.0001, Figure 2).

### 3.4. No significant correlations between LIOA and EFW or cardiac dimensions

**Figure 3:**
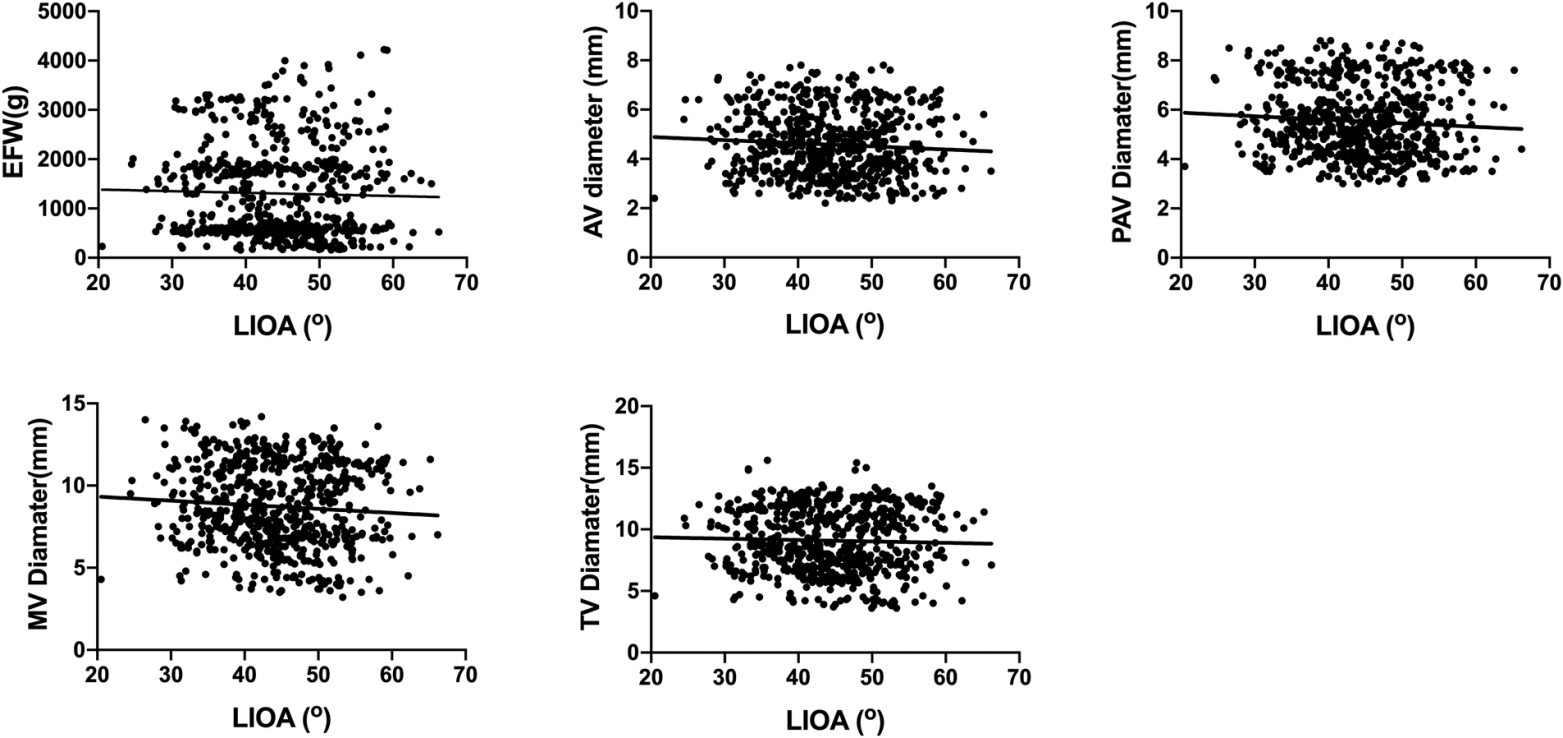
No significant correlations were found between LIOA and EFW or cardiac dimensions. The correlation between LIOA and EFW or cardiac dimensions was analyzed using Pearson's correlation coefficient. The *r* and *p*-value was *r* = −0.03, *p* > 0.05 for EFW, *r* = −0.08, *p* > 0.05 for AV diameter, *r* = −0.08, *p* > 0.05 for PAV diameter, *r* = −0.07, *p* > 0.05 for MV diameter and *r* = −0.03, *p* > 0.05 for TV diameter, respectively.

This study also investigated the correlation between LIOA and EFW as well as cardiac dimensions, including AV, PAV, MV and TV diameters. However, the findings indicated that there was no significant correlation between LIOA and either EFW or cardiac dimensions (Figure 3).

### 3.5. The intraclass correlation coefficients of inter-observer and intra-observer

The intraclass correlation coefficients of inter-observer and intra-observer for LIOA were 0.833 (95% CI, 0.721–0.910) and 0.861 (95% CI, 0.653–0.947) respectively.

## 4. Discussion

This is the first study to document the normal LIOA and explore its relationship with the cardiac axis in fetuses. We observed a consistent LIOA value of 44 ± 7.5^°^ throughout gestation (16 to 39 weeks) in normal fetuses. Additionally, a mild positive correlation was found between LIOA and the cardiac axis, while no significant correlations were found between LIOA and EFW, as well as the diameters of AV, PAV, MV and TV.

In this study, the LIOA in normal fetuses was measured to be 44 ± 7.5^°^. However, there were several studies about the supplementary angle of LIOA in adults. For instance, Kwon et al. reported a LV-aortic root angle, which is the supplementary angle for LIOA, of 140 ± 7^°^ in elderly healthy subjects. This angle was found to be decreased in patients with hypertrophic cardiomyopathy and showed an inverse correlation with age (12). Additionally, Kauhanen et al. investigated the heart-aorta-angle, also a supplementary angle for LIOA, and reported values of 127.9^°^ in patients with dilated ascending aorta and 131.9^°^ in patients with normal ascending aorta. They demonstrated that a smaller heart-aorta-angle correlated with increased total wall shear stress in the outer curvature of the proximal ascending aorta (7). The small difference observed among these angles might be attributed to variations in the age of the individual studies, as ages has been demonstrated to correlate with LV morphology (13), as well as the diameter (14) and length (15) of aorta.

In addition to our study, there was another study in fetuses on septal-aortic angle. Sternfeld et al. compared the angle between the interventricular septum and the ascending aorta between fetuses with and without cardiac anomalies (16). They reported a LVOT angle of 148.2^°^ in normal fetuses. Interestingly, they found the LVOT angle was significantly wider in fetuses with D-transposition of the great arteries (D-TGA) and valvar aortic stenosis and narrower in fetuses with complete atrioventricular canal defect. The difference between their results and ours could attribute to the different measurement methods employed. And the LVOT angle in their study for D-TGA cases was the angle with pulmonary artery not aorta. However, there were two reasons why we choose to study the LIOA in this study rather than the septal-aortic angle. Firstly, the LIOA measurement avoids interference from the morphology of septum. Additionally, the measurement method used for LIOA in our study has been shown to have high intra-observer and inter-observer reproducibility (12). Secondly, the LIOA incorporates the orientation of blood flow from LV inflow tract, which means that LIOA may have greater impacts on hemodynamics than septal-aortic angle. However, the influence of LIOA in hemodynamics needs further investigation.

Interesting, this study indicated that the LIOA angle was mildly positively correlated to cardiac axis. The cardiac axis was 40 ± 7.7^°^ in this study and was found to be independent of GA (11, 17, 18). The measurement of cardiac axis involved determining the angle between the long axis of septum and the antero-posterior axis of fetal chest, with the basal portion of septum being part of the LV outflow tract. Therefore, we speculated that alterations in cardiac axis may alter morphology of septum and LV outflow tract and then have an impact on LIOA.

There are certain limitations to this study should be acknowledged. Firstly, the investigate of LIOA was limited to the second and third trimesters, and data regarding LIOA in the first trimester were not included. Secondly, it's important to note that the sample sizes for certain GAs were relatively small, which may have influenced the generalizability of the findings.

## 5. Conclusion

In summary, this study reported a normal range of LIOA in fetuses and demonstrated its stability during the middle and third trimester. Furthermore, the LIOA was mildly positively correlated to cardiac axis and independent to EFW or cardiac dimensions, including AV, PAV, MV and TV diameters. These findings in this study may provide fundamental information for future research on aortic biomechanism in fetus with CHD.

## Data Availability

The original contributions presented in the study are included in the article/Supplementary Material, further inquiries can be directed to the corresponding author.
